# Local control after radiosurgery for brain metastases: predictive factors and implications for clinical decision

**DOI:** 10.1186/s13014-015-0367-y

**Published:** 2015-03-08

**Authors:** Tâmara Ribeiro de Azevedo Santos, Carmen Freire Tundisi, Henderson Ramos, Maria Aparecida Conte Maia, Antônio Cássio Assis Pellizzon, Maria Letícia Gobo Silva, Ricardo César Fogaroli, Michael Jenwei Chen, Sérgio Hideki Suzuki, José Eduardo Souza Dias Jr, Paulo Issamu Sanematsu Jr, Douglas Guedes de Castro

**Affiliations:** Department of Radiation Oncology, AC Camargo Cancer Center, São Paulo, Brazil; Department of Neurosurgery, AC Camargo Cancer Center, São Paulo, Brazil

**Keywords:** Radiosurgery, Brain Metastases, Whole-Brain Radiotherapy

## Abstract

**Background:**

To evaluate the local control of brain metastases (BM) in patients treated with stereotactic radiosurgery (SRS), correlate the outcome with treatment parameters and lesion characteristics, and define its implications for clinical decisions.

**Methods:**

Between 2007 and 2012, 305 BM in 141 consecutive patients were treated with SRS. After exclusions, 216 BM in 100 patients were analyzed. Doses were grouped as follows: ≤15 Gy, 16–20 Gy, and ≥21 Gy. Sizes were classified as ≤10 mm and >10 mm. Local control (LC) and overall survival (OS) were estimated using the Kaplan-Meier method. Log-rank statistics were used to identify the prognostic factors affecting LC and OS. For multivariate analyses, a Cox proportional model was applied including all potentially significant variables reached on univariate analyses.

**Results:**

Median age was 54 years (18–80). Median radiological follow-up of the lesions was 7 months (1–66). Median LC and the LC at 1 year were 22.3 months and 69.7%, respectively. On univariate analysis, tumor size, SRS dose, and previous whole brain irradiation (WBRT) were significant factors for LC. Patients with lesions >10 and ≤10 mm had an LC at 1 year of 58.6% and 79.1%, respectively (p = 0.008). In lesions receiving ≤15 Gy, 16–20 Gy, and ≥21 Gy, the 1-year LC rates were 39.6%, 71.7%, and 92.3%, respectively (p < 0.001). When WBRT was done previously, LC at 1 year was 57.9% compared with 78.4% for those who did not undergo WBRT (p = 0.004). On multivariate analysis, dose remained the single most powerful prognostic factor for LC. Median OS for all patients was 17 months, with no difference among the groups.

**Conclusions:**

Dose is the most important predictive factor for LC of BM. Doses below 16 Gy correlated with poor LC. The SRS dose as salvage treatment after previous WBRT should not be reduced unless there is a pressing reason to do so.

## Background

Brain metastases (BM) are a common outcome in the natural history of several neoplastic primary tumors, affecting up to 20–40% of patients, whether they are symptomatic or not [1]. Management of BM includes surgery, whole brain radiotherapy (WBRT), and single-dose/fractionated stereotactic radiosurgery (SRS), alone or in combination.

Historically, WBRT has been the main treatment for patients with BM [2]. In recent years, in an attempt to reduce neurotoxicity and improve local control, SRS has become an option for the management of BM as a primary treatment, either as a boost after WBRT or as a salvage treatment after WBRT failure. Two randomized trials comparing WBRT plus SRS with WBRT alone showed better local control in the SRS arm, but no significant survival benefit [3,4]. However, in a subset analysis of the Radiation Therapy Oncology Group [RTOG] 95–08 trial, the addition of SRS improved survival in selected patients who had a single BM [4].

There are no randomized trials assessing the benefit of SRS as a salvage treatment. Several small retrospective series show local control and survival at 1 year achieving rates up to 85–90% and 30–40%, respectively, rendering it an option in case of progression after WBRT [5,6]. Choosing the most appropriate treatment is still a challenge and depends on prognostic factors such as the number and size of the lesions, primary site and histological subtype of the tumor, systemic disease status, performance status, and age of the patient [7-9]. Other factors include time to intracranial progression, response to systemic treatment, symptom control, toxicity, quality of life, and consent of the patient.

Since recent improvements in systemic therapy have increased overall patient survival, local control has become an important goal of cancer treatment. Although radiosurgery is an established method for the treatment of brain metastases, data regarding factors that influence LC outcome in patients who underwent SRS are inconclusive. Moreover, dose-effect data are scarce, as most published studies follow the RTOG90–05 trial protocols, in which the dose was restricted based on toxicity with less emphasis on efficacy.

This study seeks to evaluate the local control of BM treated with SRS, correlate the outcome with treatment parameters, and define its implications for clinical decision-making.

## Methods

### Patient population

Between May 2007 and October 2012, 141 consecutive patients with 305 brain metastases were treated with SRS at a single tertiary cancer center.

From the electronic medical database, we collected patients’ characteristics including sex, age, Karnofsky Performance Status (KPS), primary site, number of lesions, presence of extracranial metastases, previous WBRT, time to recurrence after previous WBRT, recurrence after SRS, date of death or last follow-up, and cause of death. We also collected treated lesion characteristics including prescribed dose, maximum diameter, primary site, previous WBRT, radiological response, time to recurrence, and presence of radionecrosis. This study was approved by Institutional Research Board.

We excluded 68 lesions from patients with less than 3 months of clinical follow-up or those who lacked neuroimaging data after SRS, 10 lesions that underwent previous SRS or hypofractionated stereotactic radiotherapy, 6 surgical cavities, 2 small-cell lung cancer histologies, and 3 without data about dose or size. After the exclusions, 216 lesions and 100 patients were analyzed. Thirteen patients underwent multiple SRS, of which 3 patients underwent 3 treatments while 1 patient underwent 4 treatments. All lesions were included in the analysis unless the same lesion was re-irradiated.

### Radiosurgery

All patients underwent contrast-enhanced computed tomography (CT) and magnetic resonance imaging (MRI), and were immobilized using a stereotactic halo-type head frame. CT and MRI scans were co-registered in the BrainLab stereotactic planning software (BrainLab, Germany) for treatment planning. The gross tumor volume (GTV) was delineated on the CT/MRI fusion. The planning target volume (PTV) was created using a 3-dimensional volumetric expansion of 1 mm around the GTV. Patients were treated with a Varian linear accelerator based-SRS using either multiple static beams or dynamic arcs.

The dose was prescribed at the PTV margin, according to the physician’s discretion, and the prescription isodose curve varied between 90–95%. The radiation dose was loosely based on an earlier dose-escalation RTOG SRS trial (90–05) [10]. In general, we prescribed lower doses to minimize toxicity, especially for lesions near the optic pathways or brainstem. Doses were also decreased by 10% in relation to the RTOG recommendation in the event of previous WBRT, in order to keep the risk for late neurological sequelae after SRS lower than 10%.

### Follow-up

Patients received a follow-up MRI every 1–3 months during the first year after SRS. Thereafter, additional brain imaging was done based on neurologic symptoms or as part of a routine clinical follow-up by the treating radiation oncologist, clinical oncologist, or neurosurgeon.

### Response evaluation

Tumor response was evaluated based on any change in the size of the tumor on serial MRI scans obtained after the completion of SRS, and on the reviewing of patient records. Complete disappearance of the tumor was defined as a complete response (CR), a decrease in tumor size was defined as partial response (PR), no change in tumor size was defined as stable disease (SD), and a non-transitory increase in the size of the tumor was defined as progressive disease (PD). In some cases, the determination of the response was reached after MRI spectroscopy and perfusion imaging. None of the PD cases were confirmed by tissue histology.

The objective response rate was based on the combined number of lesions designated as CR or PR. Non-responders were defined as patients with SD or PD lesions. LC rate was defined as the rate of lesions with CR, PR, and SD.

### Statistical analyses

Doses were grouped as follows: ≤15 Gy (17.1%), 16–20 Gy (59.3%), and ≥21 Gy (23.6%). Tumor size was classified as ≤10 mm (53.7%) and >10 mm (46.3%). The primary sites of the lesions were breast (37.5%), lung (28.2%), melanoma (26%), and other sites (8.3%).

The actuarial LC was calculated according to the Kaplan-Meier method from the date of SRS to the date of last MRI (if CR, PR, or SD) or to the date of the MRI that showed progression. Overall survival (OS) was calculated from the day of SRS until the date of death or last follow-up using the Kaplan-Meier method. We used a significance level of 5% (p value ≤ 0.05).

Log-rank statistics were used to identify the prognostic factors affecting the LC. For multivariate analyses, a forward stepwise approach using a Cox proportional model was applied, including all potentially significant variables reached on univariate analyses.

## Results

Among the 100 patients evaluated, the majority were women (63%), the median age was 54 years (range: 18–80), and the median KPS was 90% (range: 60–100). Seventy percent of patients had metastases outside the brain, 39% had undergone previous WBRT, the median number of simultaneously treated lesions was 2 (range: 1–11), and the median follow-up time was 11 months (3–66) (Table 1).

**Table 1:** **Patient and tumor characteristics**

**Patient Characteristics**	**Incidence (%)**
**Primary Site**	
Breast	32 (32%)
Lung	30 (30%)
Melanoma	26 (26%)
Others	12 (12%)
**KPS**	
≥60-70%	1 (1%)
70-80%	35 (35%)
90-100%	64 (64%)
**Extracranial systemic disease**	
Yes	70 (70%)
**Number of Lesions**	
1	48 (48%)
2	22 (22%)
3	12 (12%)
≥4	18 (18%)
**Lesion Characteristics**	**Incidence (%)**
**Size (mm)**	
≤10	116 (53.7%)
>10	100 (46.3%)
**Primary site**	
Breast	81 (37.5%)
Lung	61 (28.2%)
Melanoma	56 (26%)
Others	18 (8.3%)
**Dose (Gy)**	
≤15	37 (17.1%)
16-20	128 (59.3%)
≥21	51 (23.6%)
**Previous WBRT**	
Yes	95 (44%)
No	121 (56%)

Regarding lesion characteristics (Table 1), the prescribed dose ranged from 12 Gy to 24 Gy (median: 18 Gy) and tumor sizes ranged from 2 to 31 mm (median: 10 mm). In 95 of the 216 lesions analyzed (44%), the patient had been previously treated with WBRT, 6 as a boost and 89 for salvage purposes. The median dose in patients that had or had not previously received WBRT was 16 Gy (range: 12–20) and 20 Gy (range: 13–24), respectively. The median interval between WBRT and SRS was 10 months (range: 1–28). In the remaining 121 lesions (56%), the SRS was delivered as a primary treatment.

### Local control and predictive factors

The median radiological follow-up time of the lesions was 7 months (range: 1–66). The median LC and the LC at 1 year were 22.3 months and 69.7%, respectively.

On univariate analysis, tumor size, SRS dose, and previous WBRT were significant for progression. LC rate at 1 year was not different when considering the primary site: breast (64.5%), lung (79%), melanoma (72.3%) and other (54.6%) (p = 0.06) (Table 2).

**Table 2:** **Tumor response according to dose**

**Local Control**	**1 year (%)**	**Median (months)**	**CI**	**p value**
Global	69.7	22.3	18.7–25.9	-
**Size**				
≤10	79.1	24.6	-	0.008
>10	58.6	20	9.5–30.5	
**Primary Site**				
Breast	64.5	20	11.4–28.6	0.06
Lung	79	NR	-	
Melanoma	72.3	NR	-	
Others	54.6	12.2	5.2–9.3	
**Dose (Gy)**				
≤15	39.6	10.8	7.7–3.9	<0.0001
16–20	71.7	24.6	18.9–30.2	
≥21	92.3	NR	-	
**Previous WBRT**				
Yes	57.9	13.7	4.8–22.5	0.004
No	78.4	NR	-	

Patients with lesions >10 mm had worse outcomes, with an LC rate at 1 year of 58.6% and a median LC of 20 months. In contrast, for lesions up to 10 mm, the LC rate at 1 year was 79.1% and the median LC was 24.6 months (p = 0.008).

**Figure 1:**
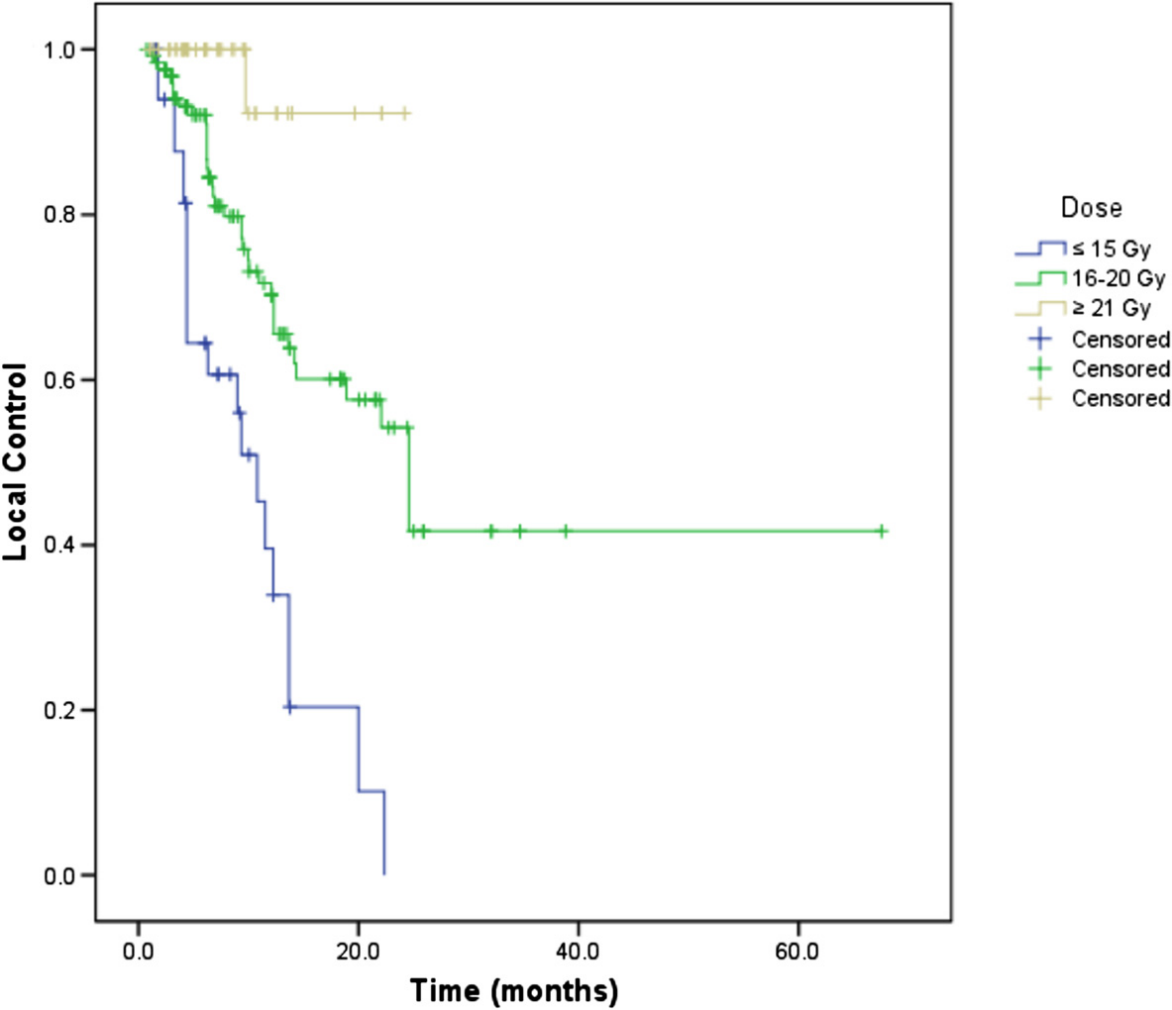
**Kaplan-Meier curve for local control by dose groups.**

In lesions receiving ≤15 Gy, 16–20 Gy, and ≥21 Gy, the 1-year LC rates were 39.6%, 71.7% and 92.3%, respectively. The median LCs were 10.8 and 24.6 months for lesions receiving ≤15 Gy and 16–20 Gy, respectively, and were not achieved for doses above 21 Gy (p < 0.001) (Figure 1).

**Figure 2:**
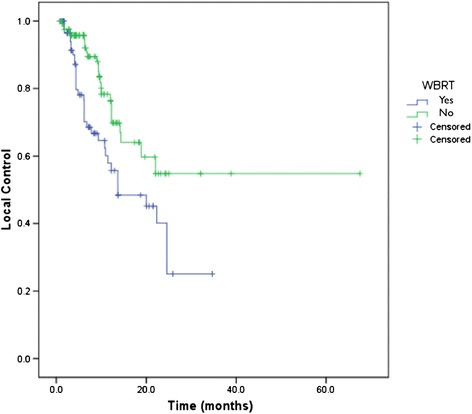
**Kaplan-Meier curve for local control by previous whole brain irradiation status.**

When patients had previously undergone WBRT, the LC at 1 year was 57.9% compared with 78.4% for those who did not receive WBRT (p = 0.004) (Figure 2).

**Figure 3:**
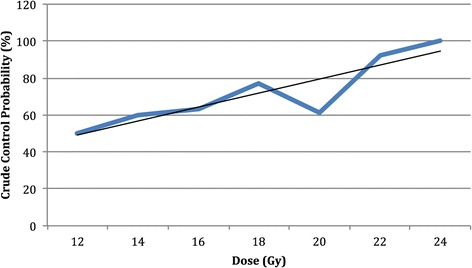
**Dose–response curve with trend line.**

An objective response was observed in 82 of 216 lesions (response rate, 38%): 27 lesions achieved a complete response (12.5%) and 55 had a partial response (25.5%). Progression occurred in 60 lesions (27.8%) while 74 lesions were stable (34.3%). The objective response rates according to doses of ≤15 Gy, 16–20 Gy and ≥21 Gy were 10.8%, 33.6%, and 68,6%, respectively. A dose–response curve was generated from our data (Figure 3).

After SRS, 63 patients (63%) experienced recurrence, 11% of them in the treated lesions only, 31% in other sites of CNS, and 21% in both. The median interval to first recurrence was 4 months (range: 0.6–25 months). Five of the 6 lesions that received SRS as a boost after planned WBRT recurred.

On multivariate analysis, dose remained the single most powerful prognostic factor for the LC of brain metastases (Table 3).

**Table 3:** **Multivariate analysis**

**Factor**	**Estimate**	**p value**	**HR**	**95% CI**
Dose		0.0021		
≤15 *versus* ≥21	3.10	0.0036	22.21	[2.76; 178.56]
16–20 *versus* ≥21	2.22	0.0305	9.18	[1.23; 68.49]
Size (≤10 *versus* >10) mm	−0.47	0.1127	0.63	[0.35; 1.12]
WBRT (Yes versus No)	0.09	0.7877	1.09	[0.58; 2.05]

### Overall survival

The median OS for all the patients was 17 months. When stratified by doses, there was no difference among the groups. The OS at 6 months, 1 year, and 2 years was 84%, 66% and 41%, respectively. Fifty-one patients (51%) died of cancer, 11 of whom died from progression in the CNS. Thirty-nine patients are still alive. We lost follow-up contact with 10 patients.

### Toxicity

In this analysis, 10 lesions developed radionecrosis of which only 2 became symptomatic, one needing routine corticosteroids and the other requiring surgery. Five of the patients that developed necrosis had previous WBRT and were treated with doses between 14–19 Gy. The other 5 lesions had no prior WBRT: 4 lesions received 21 Gy (all of which were asymptomatic) and 1 was treated with 16 Gy.

## Discussion

Since recent improvements in systemic therapy, LC of BM has become an important issue. Several trials indicate that SRS plus WBRT improve the LC of BM compared to WBRT alone [3-11]. Nevertheless, higher doses increase brain necrosis [10].

The ideal dose of SRS has not been established. Most institutions prescribe doses based on the RTOG 90–05 trial, which was a study of toxicity and not efficacy. This prospective study established a maximum SRS dose of 24 Gy for lesions of less than 2 cm, 18 Gy for lesions between 2–3 cm, and 15 Gy for lesions between 3–4 cm in patients with previously irradiated brains.

Several retrospective studies have published data around factors affecting the LC of BM with SRS. In most series, the 1-year LC rate is higher than 80% for doses above 20 Gy, and higher than 60% for doses of 18 Gy. For lesions treated with doses below 15 Gy, the 1-year LC rate is poor, at less than 50%. The LC at 6 months is high in most series, usually above 80%, independent of the dose.

Vogelbaum et al. reported a significantly better LC at 1 year in tumors receiving 24 Gy (85%) compared to lesions treated with 18 Gy (49%) or 15 Gy (45%). There was no difference in LC between lesions receiving 18 or 15 Gy [12]. Shehata et al. evaluated the efficacy of the SRS dose exclusively in tumors ≤20 mm, whether or not they were associated with planned WBRT. They found that doses ≥20 Gy were significantly more effective than doses below 20 Gy, with LC rates of 99% and 91%, respectively (p = 0.0029). Planned WBRT associated with SRS also increased the LC compared to SRS alone (97% vs. 87%; p = 0.0001). When doses >20Gy were prescribed after WBRT, there was higher toxicity with no improvement in the LC, suggesting that 20 Gy is the optimal dose after planned WBRT [13]. On multivariate analysis, Schomas et al. found that the minimum tumor dose (TDmin) is the only predictive factor of LC after controlling for histology, volume, and prescription dose. The actuarial 1-year LC was 66.7% with a TDmin ≤12 Gy, which was inferior to lesions treated with a higher TDmin (>93%) [14].

Chao et al. reported the efficacy of SRS as a salvage treatment in a study involving 111 patients at the Cleveland Clinic. The LC rate at 1 year was 68%. There was significantly better LC at 1 year in tumors receiving ≥22 Gy compared with those receiving <22 Gy (92% and 72% respectively). Considering BM ≤2 cm, the 1-year LC was 91% versus 62% of lesions >2 cm (p < .0001) [15]. Similarly, Chang et al. prescribed 20–24 Gy for all brain metastases and found better LC rates for lesions <1 cm compared to those >1 cm (86% vs. 56%, respectively) [16].

Our results are similar to most published studies. In our series, the only significant factor that predicted LC was dose, with BM treated with ≥21 Gy achieving better control than groups receiving 16–20 Gy or ≤15 Gy. There was also a difference in control between doses of 16–20 Gy and ≤15 Gy. We also found a difference in control based on the maximum diameter of the lesion on univariate analysis, but this contrast was not detected on multivariate analysis (Table 4).

**Table 4:** **Comparative series**

**Author, year**	**Patients/ Lesions**	**Treatment options**	**Dose**	**Local Control (LC)**	**Predictive factors**	**Complication (%)**
Noel G, [5]	92/ 145	SRS alone: 34 (37%)	**WBRT**: 30Gy/10 or 40Gy/20	**6-months:** 93% **1-y:** 86%	high maximal dose delivered at the isocentre of the GTV	8 - radiation necrosis (5.5%)
		SRS + WBRT: 22 (24%)	**SRS alone:** 14Gy*	SRS alone:		1 - seizure
		WBRT + salvage SRS: 36 (39%)	**SRS plus WBRT:** 10Gy*	**6-m:** 90% **1-y:** 78%		
			* On 70% isodose line	SRS + WBRT: **6-m/1-y:** 90%		
				WBRT + SRS:		
				**6-m:** 92% **1-y:** 86% (NS)		
Noel G, [6]	54/ 97	WBRT + salvage SRS	**WBRT:** 30-40Gy	**1-y:** 91.3% **2-y:** 84%	no prognostic factors for local control	no major complications
			**SRS**			
			minimun dose: 14.4Gy (12.3-19.3)			2 - transient headaches secondary to edema
			maximum dose: 20.9 Gy (17.3-38.8)			2 - temporary grade 2 alopecia
Vogelbaum, [12]	202/ 375	SRS alone: 48 (24%)	≤20 mm: 24Gy	**median:**	dose to the tumor margin	6 - proven radiation necrosis
		SRS + WBRT: 37 (18%)	21-30 mm: 18Gy	24Gy: NR** 18Gy: 11,57 m 15Gy: 11,83 m		
		WBRT + salvage SRS: 117 (58%)	31-40 mm: 15Gy	**1-y** 24Gy: 85%** 18Gy: 49% 15Gy: 45%		
			* At tumor margin (50% isodose line), independent if previous WBRT or not	** for 24 Gy compared with 18 or 15 Gy		
Shehata, [13]	160/ 468	SRS alone: 228 mets (49%)	planned **WBRT**: 6.75– 50.4 Gy (median 40.5 Gy)	**Overall LC**	addition of WBRT	trend toward greater complications (RTOG Grade 3 or 4) for SRS doses >20 × ≤20 Gy (p = 0.078)
		SRS + planned WBRT: 240 mets (51%)	**SRS:** 7-30Gy at the 40–95% isodose (median 60%)	SRS alone: 87% SRS + WBRT: 97%	If planned WBRT - dose	
			maximal dose:10.7-50 Gy (median 30 Gy)	**1-y**	tumor volume	
				SRS alone: 77% SRS + WBRT: 96% **		
				SRS < 20 Gy + WBRT: 91%		
				SRS ≥ 20 Gy + WBRT: 99%**		
				If 20Gy: 99% >20Gy: 96% (NS)		
Schomas, [14]	80/ 126	SRS alone: 11 (14%)	**WBRT:** 25–46 Gy in 2–3 Gy	**1-y:** 88.6%	minimum target dose	3 patients (5%)
		SRS + planned WBRT: 69 (86%)	**SRS:** 18 Gy (10–21)	SRS + WBRT: 88.8%		2 - edema
				SRS alone: 87.5% (NS)		1 - radiation necrosis (confirmed by resection)
				**Per dose** ** ≤ 12Gy: 66.7%		2- within SRS field
				12.1-18Gy: 93.8%		1- outside field (previous WBRT)
				>18Gy: 93.3%		
				**2-y:** 77.2%		
Chao, [15]	111/ NA	WBRT + salvage SRS	**WBRT:** 37.5 Gy (30–50)	**1-y:** 68%	tumor size dose	2 - radiation necrosis (5,5 months and 1.5 year after SRS)
			**SRS:**	**2-y:** 59%		1 - seizure
			≤20 mm: 24Gy 21-30 mm: 18Gy			
			31-40 mm: 15Gy			
			>40 mm: 12Gy			
Chang, [16]	135/ 153	SRS alone: 71 (52,6%)	**WBRT:** 30Gy (22.5-40Gy)	**1-y:** 69%	tumor volume cone diameter	edema with mass effect (14%)
		SRS + WBRT: 30 (22,2%)	**SRS**	≤1 × > 1 cm: 86 × 56%**		Pathologically proven necrosis (1,3%)
		WBRT + salvage SRS: 32 (23,7%)	Minimum peripheral dose: 20-24Gy	**2-y:** 46%		Hemorrhage (4,6%)
				≤1 × > 1 cm: 78 × 24%**		
Our series, 2015	100/216	SRS alone: 121 (56%)	**WBRT:** 30 Gy (median)	**1-y:**	dose at the PTV margin	8 – asymptomatic radiation necrosis
		WBRT + salvage SRS: 95 (44%)	**SRS alone:** 20 Gy (median)	SRS alone: 78.4%		2 – symptomatic radiation necrosis
			**WBRT + salvage SRS:** 16 Gy (median)	WBRT + SRS: 57.9%**		
				Per dose**:		
				≤15 Gy: 39.6%		
				16-20Gy: 71.7%		
				≥21 Gy: 92.3		

Legends: * isodose prescription; ** statistically significant; NS: not significant; NA: not available.

It is difficult to compare our results to those of other studies because others reported LC based in the tumor volume or used different cut-off sizes. The results are variable, with some investigators reporting better LC with smaller volumes on multivariate analysis and others reporting no difference. It is important to note that size and SRS dose are highly correlated, since higher doses are usually prescribed for smaller reasons.

Prior WBRT was a significant factor for LC on univariate analysis in this study. Since the multivariate analysis showed only dose as a predictive factor, we can attribute this loss of control due to the reduction of the dose of SRS when performed as a salvage treatment after WBRT at our institution.

Not all institutions reduce the dose after WBRT, and some reduce it in the boost setting. In his seminal study, Aoyama (based on his prior experience) reduced doses by 30% if a planned WBRT was administered before SRS. In this study, both the control of the lesion and CNS were higher than SRS alone. The prescribed doses were 22–25 Gy for lesions up to 2 cm and 18–20 Gy for lesions larger than 2 cm. Their actuarial local tumor control rate at 12 months was higher for the WBRT + SRS arm. Only 1 patient in the SRS alone group and 2 in the WBRT + SRS groups presented with grade 4 radionecrosis [11].

Centers that lower the dose of salvage SRS after WBRT generally do so for fear of possible toxicities. Nevertheless, studies show a low rate of radiation necrosis even after a previous course of WBRT. The RTOG 95–08 trial followed the RTOG 90–05 guidelines for doses for SRS boost after planned WBRT. Six percent of patients in the SRS arm developed grade 3 or 4 toxicity (radionecrosis not specified). Radionecrosis grade 3 or 4 was reported in the RTOG 90–05 study, in which there was an 8% radionecrosis rate at 12 months. As reported in some studies, most incidents of radionecrosis are asymptomatic or can be controlled with corticosteroids [4,10].

In a systematic review, Wiggenraad et al. found that 1-year LC rates varied and were higher than 80%, higher than 60%, and lower than 50% with single doses of ≥21 Gy, ≥18 Gy, and ≤15 Gy, respectively. One-year LC rates were 70% or higher with fractionated stereotactic radiotherapy (FSRT), since the biological effective dose (BED), with an α/β of 12 Gy, was at least 40 Gy [17].

Therefore, following the evidence in the literature and our institutional experience, we have been optimizing the patient selection for SRS. In extreme situations, like large lesions or those adjacent to critical structures that would limit the SRS dose to <16 Gy, we prefer to perform FSRT with an adequate BED in order to offer a high probability of LC with an acceptable toxicity. Otherwise, we are no longer lowering SRS doses for patients with previous WBRT.

This study has some limitations beyond its retrospective biases. Only one patient had pathologic confirmation of radionecrosis, and since the distinction between progression and necrosis is difficult with imaging modalities, some lesions that were considered progression may have been necrosis, and vice-versa. We tried to limit the probability of this error by using MRI with perfusion and spectroscopy in questionable cases, and also retrospectively analyzing the MRIs in case any lesion decreased in size after an initial increase. Otherwise, we did not analyze when or whether systemic treatment was used, which could be a confounding factor since it is well known that some systemic agents, mainly target drugs, have some effect on the CNS. Some patients received lower doses than they otherwise should have because their tumors were located in or near critical structures, or if they had previously received high doses of radiotherapy. It should be noted that toxicity was defined in our study solely on the basis of radiographic follow up; there was no assignment of RTOG CNS toxicity grades in our report.

## Conclusion

This study suggests that dose is the most important predictive factor for the LC of brain metastases. Doses below 16 Gy correlated with poor LC. The SRS dose as salvage treatment after previous WBRT should not be reduced unless there is a pressing reason to do so.

## Abbreviations

BM: Brain metastases
SRS: Stereotactic radiosurgery
LC: Local control
OS: Overall survival
WBRT: Whole-brain irradiation
RTOG: Radiation Therapy Oncology Group
KPS: Karnofsky performance status
CNS: Central nervous system
CT: Computed tomography
MRI: Magnetic resonance imaging
GTV: Gross tumor volume
PTV: Planning target volume
CR: Complete response
PR: Partial response
SD: Stable disease
PD: Progressive disease
TDmin: Minimum tumor dose
FSRT: Fractionated stereotactic radiotherapy
BED: Biological effective dose
